# All-trans retinoic acid reduces cancer stem cell-like cell-mediated resistance to gefitinib in NSCLC adenocarcinoma cells

**DOI:** 10.1186/s12885-020-06818-0

**Published:** 2020-04-15

**Authors:** Wenxiu Yao, Liyang Wang, Huan Huang, Xin Li, Pinjia Wang, Kun Mi, Jia Cheng, Huifen Liu, Cuirong Gu, Lingxiao Huang, Jianming Huang

**Affiliations:** 1 Department of Medical oncology, Sichuan Cancer Hospital, Medical School of University of Electronic Science and Technology of China, Chengdu, 610041 People’s Republic of China; 2 Department of Medical oncology, Wuming Hospital Affiliated to Guangxi Medical University, Nanning, 530000 China; 3 Department of Biochemistry & Molecular Biology, Sichuan Cancer Insititute, No.55, Section 4, South Renmin Road, Chengdu, 610041 People’s Republic of China

**Keywords:** All-trans retinoic acid, ALDH1A1, CD44, EGFR tyrosine kinase inhibitors, Non-small cell lung adenocarcinoma

## Abstract

**Background:**

The enrichment of cancer stem cell-like cells (CSCs) has been considered to be responsible for tumor progression after an initial response to epidermal growth factor receptor (EGFR) tyrosine kinase inhibitors (EGFR-TKIs) in patients with non-small cell lung adenocarcinoma (NSCLC/ADC). CSCs with ALDH1A1^bright^ /CD44^high^ expression contribute to the TKIs resistance in NSCLC/ADC cells. All-trans retinoic acid (ATRA) has been shown to be a potential targeted therapy against CSCs due to its ability to inhibit ALDH1A1 activity. We therefore investigated whether ATRA could circumvent the resistance to improve the response to gefitinib in NSCLC/ADC cells.

**Methods:**

Treatment of NSCLC/ADC A549 and H1650 cells with gefitinib enriched the gefitinib surviving cells (GSCs). The expression of ALDH1A1 and CD44 and the IC50 values for gefitinib were determined by flow cytometry (FCM) and crystal violet assay in GSCs and ATRA-treated GSCs, respectively. Using DEAB as the positive control, direct inhibitory effect of ATRA on ALDH1A1 activity was determined by ALDEFLUOR assay,

**Results:**

GSCs showed higher expression of ALDH1A1 and CD44 and IC50 values for gefitinib than their respective parental cells, suggesting that gefitinib can lead to propagation of CSC-enriched gefitinib-resistant cells. Treatment with ATRA was found to significantly reduce the increased expression of ALDH1A1 and CD44 and the IC50 values for gefitinib in A549GSC and H1650GSC cells, and ATRA could directly inhibit active ALDH1A1 as compared to DEAB.

**Conclusion:**

Our findings suggest that combination treatment with ATRA prevents gefitinib-induced enrichment of ALDH1A1^bright^/CD44^high^ CSCs and enhances gefitinib-induced growth inhibition of NSCLC/ADC cells.

## Background

Non-small cell lung cancer (NSCLC) is the most common type of lung cancer, accounting for 84% of all lung cancer diagnoses [1]. For advanced NSCLC, median survival remains poor at 7.9 months and only approximately one third of patients survive for 1 year or more despite conventional combination chemotherapy [2]. About 10 and 30% of NSCLCs in Western and Asian populations, respectively, express an activated mutant epidermal growth factor receptor (EGFRm) and the majority of such patients respond to adenosine triphosphate (ATP)-competitive EGFR tyrosine kinase inhibitors (EGFR-TKIs) such as gefitinib [3–5].

Despite initial dramatic efficacy of EGFR-TKIs in NSCLC patients with or without EGFR mutation, emergence of acquired resistance is almost inevitable at a median of 9–13 months, thereby limiting the benefits of EGFR-TKIs to NSCLC patients’ outcomes [5–8]. EGFR-TKIs resistance has become a major clinical challenge in NSCLC.

EGFR T790M-mutation-mediated TKIs resistance account for about 50% in acquired resistance of NSCLC patients, and the remaining 50% of patients acquire resistance to EGFR-TKIs via a non-T790M mediated resistance mechanism [9–11]. Alterations including secondary EGFR T790M mutation, MET amplification, and appearance of EMT features were observed in NSCLC. Cancer drug resistance has recently been linked, at least in part, to a small sub-population of cells within the tumour that possess stem-like properties [12]. It has been demonstrated that stem cell-like properties are enriched in CD44^high^ and ALDH1A1^bright^ subpopulations of some NSCLC cell lines [13, 14]. However, the manner of EGFR-TKIs exposure influences the mechanism of acquired resistance and the appearance of stem cell–like property with EGFR-TKI treatment. After initial gefitinib treatment, the residual cancer cells can survive the initial cycles of treatment and are proved to be a subset of gefitinib-induced enrichment of cancer stem-like cells (CSCs) [12]. It has been reported that the high-concentration gefitinib-exposure methods revealed different resistance mechanisms and CSC properties with ALDH1A1 and CD44 overexpression [13, 14]. Cancer stemness induced via up-regulation of ALDH1A1 and CD44 expression contributes to the acquisition of gefitinib resistance in EGFR-TKI sensitive NSCLC [15]. CSCs with ALDH1A1^bright^ and CD44^high^ may play a significant role in acquired resistance to gefitinib [16, 17]. A study reported that treatment of lung cancer cell lines with TKIs enriches the ALDH^bright^ stem-like cells through EGFR-dependent activation of Notch3 and inhibition of EGFR kinase activity leads to an increase in ALDH^bright^ cells [18]. It has been documented that inhibition of ALDH1A1 activity reduces chemotherapy resistance in various cancers such as ovarian and breast cancers [19–22]. As is reported, ALDH1A is linked to retinoic acid signaling and all-trans retinoic acid (ATRA), as a potential targeted therapy against cancer stem cells, could inhibit ALDH1A1 activity to improve chemotherapeutic efficacy [23, 24]. However, the role of ATRA in NSCLC is less well understood, in part, since it has not been fully established which cell subpopulation express the retinoic acid receptors (RARs). Studies have confirmed that RAR and RXR are expressed in NSCLC CSCs that co-express ALDH1 [25–27]. Using therapy-induced enrichment of CSCs may, therefore, prove to be an extremely useful method for studying CSCs and provide new clues regarding potential therapeutic targets such as ALDH1A1 for their efficient elimination, which will undoubtedly play an indispensable role in improving NSCLC patients’ survival.

In this study, we explored whether ATRA reduces resistance of therapy-induced enrichment of NSCLC CSCs through inhibiting ALDH1A1 activity to circumvent gefitinib resistance in NSCLC/ADC.

## Methods

### Cell culture and reagents

The human NSCLC/ADC H1650 cell line with EGFR^delE746-A750^ mutation and A549 cell line with EGFR^wt^ were obtained from the Committee on Type Culture Collection of Chinese Academy of Sciences (CTCCCAS, Shanghai, China). H1650 and A549 cells were chosen because their response to gefitinib have been extensively characterized (primary resistance to gefitinib).

Cell lines were cultured in RPMI 1640 medium (GIBCO) that contained 10% fetal calf serum (FCS), 2 mmol/L L-glutamine and 100 units/mL penicillin and 40 IU/mL gentamycin were maintained at 37 °C in a humidified atmosphere of 5% CO_2_ and 95% air. Subconfluent cells (80%) were passaged with a solution containing 0.25% trypsin-0.5 mmol/L Ethylenediaminetetraacetic acid (EDTA). Cell lines were tested for mycoplasma and confirmed to be negative.

Gefitinib (N-[3-Chloro-4-fluorophenyl]-7-methoxy-6-[3-morpholinopropoxy]quinazolin-4- amine, CAS 184475–35-2, MF C_22_H_24_ClFN_4_O_3_, MW 446.907 g/mol, HPLC> 98%) and all-*trans* retinoic acid (ATRA, [2E,4E,6E,8E]-3,7-dimethyl-9-[2,6,6-trimethylcyclohexen- 1-yl] nona-2,4,6,8-tetraenoic acid, CAS 302–79-4, MF C_20_H_28_O_2_, MW 300.442 g/mol, HPLC> 98%) were purchased from Sigma Chemical Co. (St. Louis, MO, USA); ALDEFLUOR™ Kit (Cat. No.01700) was purchased from STEMCELL Technologies Inc.; BD Pharmingen™ PE mouse anti-human CD44 monoclonal antibody (Clone 515 Cat No. 550988) and its isotype mouse BALB/c IgG1 were purchased from BD Biosciences (Lake Franklin, NJ, USA).

AmoyDx ARMS EGFR mutation detection kit was purchased from Amoy Diagnostics Co. LTD (Xiamen, China).

### Cell viability assay

Cell viability was measured by a colorimetric assay using crystal violet. To a 96-well plate, 5 × 10^3^ cells/well were pre-cultured for 24 h, and then exposed to varying concentrations of gefitinib and ATRA, and 0.1% DMSO was used as a vehicle in triplicate. After 72 h, the supernatant was discarded as much as possible, and 100 μL of crystal violet solution (0.5% crystal violet in 30% methanol) was added to each well for 30 min, and then rinsed with tap water and dried at 40 °C. 100 μL of 10% SDS solution was added to each well and fully dissolved for 30 min. The absorbance at 595 nm was measured spectrophotometrically using a microplate reader (Infinite M200 Pro TECAN-Reader, Switzerland).

### EGFR mutation testing

Genomic DNA from A549 and H1650 cells was manually extracted using a TIANamp Genomic DNA Kit (DP304, TIANGEN, China.) according to the manufacturer’s protocol. DNA was isolated by elution with 50 μl of Tris/Acetate/EDTA (TAE). EGFR mutations were detected with the AmoyDx Human EGFR Gene 29 Mutations Detection kit with fluorescence polymerase chain reaction (PCR) (Amoy Diagnostics, Xiamen, China) and assays were performed on CFX96 Touch (Bio-Rad, USA) real-time fluorescence quantitative PCR instrument according to the manufacturer’s instructions. Positive results were defined as [*Ct* (sample)-*Ct* (control)] \ *Ct* (cut-off).

### Gefitinib-induced enrichment of GSC and ATRA treatment

H1650 and A549 cells were passaged with 15 μmol/L of gefitinib twice weekly for three consecutive weeks, and the resultant gefitinib surviving cells (A549GSC cells and H1650GSC cells) were incubated with 5 μmol/L of ATRA for 1–5 days. These cells were respectively harvested to test the expression of ALDH1A1 and CD44 by flow cytometer (FCM). The GSCs with enhanced expression of ALDH1A1 and CD44 are defined as GSC-enriched gefitinib-resistant cells**.**

### Flow cytometry for ALDH1A1 and CD44 expression

Expression of ALDH1A1 and CD44 by A549 and H1650 cells were determined using ALDEFLUOR™ kit (FITC) and CD44 mAb (PE), respectively according to the manufacturer’s protocols. Briefly, A549 and H1650 cells (1 × 10^6^) were harvested and stained with ALDH (DEAB as the negative control) and PE anti-human CD44 mAb (mouse IgG1 as the isotype control) staining. The stained cells were resuspended in 1 ml of Assay Buffer and subjected respectively to flow cytometrical analysis on FACSCanto II Flow Cytometer (Becton–Dickinson).

### Determination for inhibition of ATRA on ALDH1A1 activity

Active ALDH1A1 was determined using ALDEFLUOR assay according to the manufacturer’s protocol. A549 GSCs and H1650 GSCs with ALDH1A1^bright^ (5 × 10^5^ cells/tube) were respectively exposed to varying concentrations of ATRA and DEAB (diethylaminobenzaldehyde, an inhibitor of ALDH1A1 activity), and washed twice with 2 ml ALDEFLUOR buffer and eventually resuspended in 500 μl ALDEFLUOR buffer, and then subjected to flow cytometrical analysis to determine the FITC AUC (area under curve) on FACS Canto II Flow Cytometer (Becton–Dickinson).

## Results

### Growth inhibition of H1650 and A549 cells by gefitinib and ATRA

**Fig. 1:**
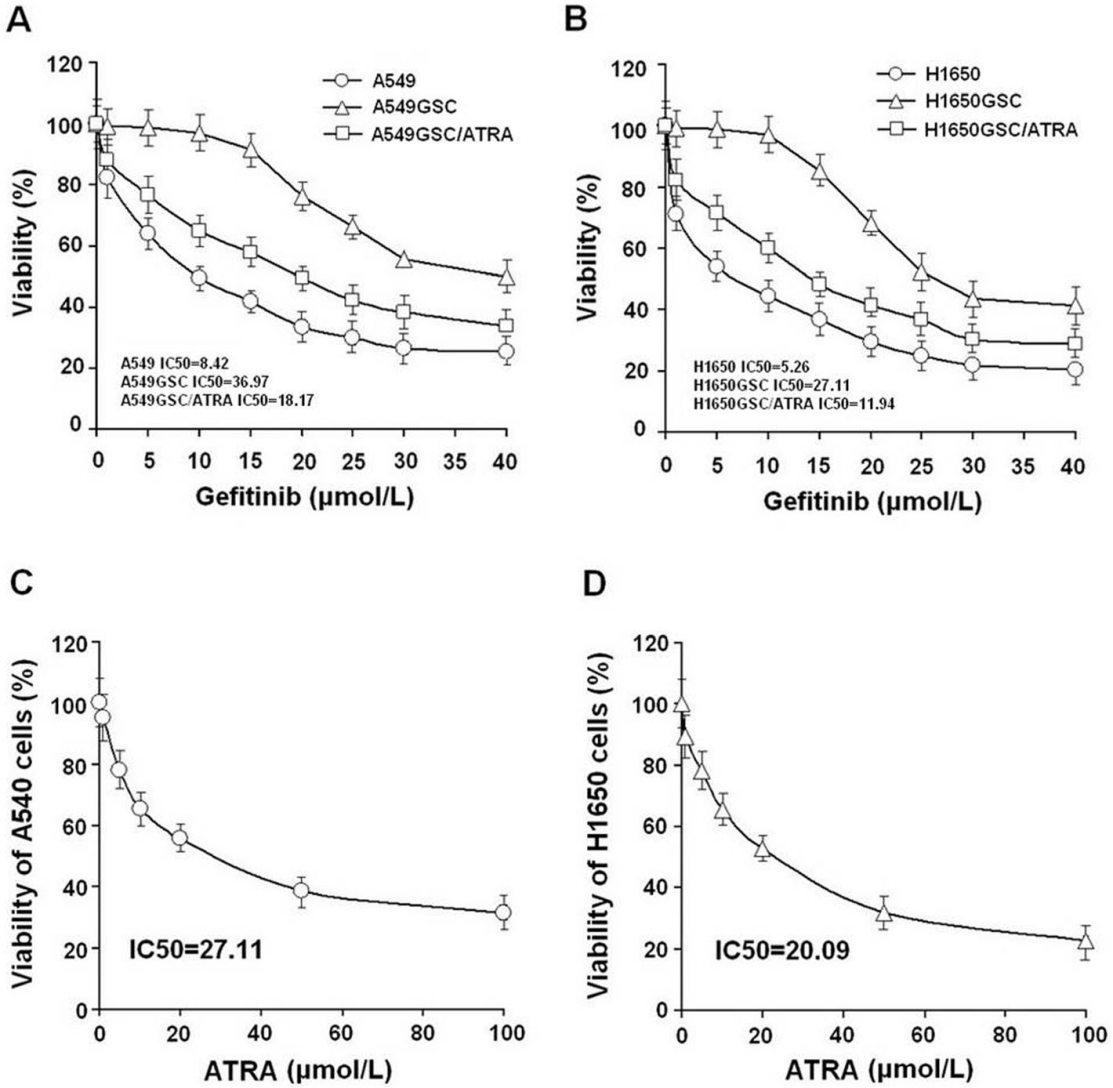
Growth inhibition of gefitinib and ATRA on lung cancer cells. **a**. Cell viability (%) of A549 and A549GSC cells treated with gefitinib alone or combined with 10 μmol/L ATRA for 72 h; B. Cell viability (%) of H1650 and H1650GSC cells treated with gefitinib alone or combined with 10 μmol/L ATRA as described in Methods. **c** and **d**. Cell viability (%) of A549 and H1650 cells treated with ATRA alone, respectively. All experiments were performed in triplicate, and data are expressed as means±s.d. (*n* = 3). Error bars represent s.d. of replicate data points. *P < 0.05, compared to the control

As shown in Table 1 and Fig. 1a-d, we showed that there was no significant difference between H1650 cells and A549 cells for the response to gefitinib (IC50 5.26 vs. 8.42 μmol/L), however the IC50 values of gefitinib for H1650GSC and A549GSC cells significantly increased by 5.15-fold (from 5.26 to 27.11 μmol/L) and 4.39-fold (from 8.42 to 36.97 μmol/L), respectively as compared to their untreated cells (both *P* < 0.01). We found that pre-incubation with ATRA significantly enhanced gefitinib-induced growth inhibition and decreased the IC50 values for gefitinib by up to 2.27-fold (from 27.11 to 11.94 μmol/L) (*P* < 0.01), and 2.04-fold (from 36.97 to 18.17 μmol/L) (*P* < 0.01) for H1650GSC and A549 GSC cells, respectively. Interestingly, we found that ATRA significantly inhibited the growth of H1650GSC and A549GSC cells but did not obviously impact the growth of H1650 and A549 cells. These results suggest that both H1650GSC and A549GSC cells have a higher resistance to gefitinib, and ATRA could improve the response of H1650GSC and A549GSC cells to gefitinib.

**Table 1:** Gefitinib-induced growth inhibition in combination with ATRA in NSCLC/ADC cells

Cell line	*n*	Gefitinib		Gefitinib plus ATRA treatment	
		IC50 (μmol/L)	*P*	IC50 (μmol/L)	*P*
A549	3	8.42 ± 0.312	–	–	–
A549GSC	3	36.97 ± 2.017	< 0.01	18.17 ± 1.271	< 0.01
H1650	3	5.26 ± 0.204	–	–	–
H1650GSC	3	27.11 ± 1.891	< 0.01	11.94 ± 1.153	< 0.01

NSCLC/ADC cells were treated with vehicle or gefitinib and treated with ATRA (10 μmol/L) plus gefitinib for 72 hoursh. Relative number of viable cells was assayed using crystal violet assay. IC50 values were measured as a curvilinear regression equation for each survival curve. Data represent mean values ± S.D. from three independent experiments. *P values, compared with gefitinib alone

### GSC-enriched cell population mediates gefitinib resistance

**Fig. 2:**
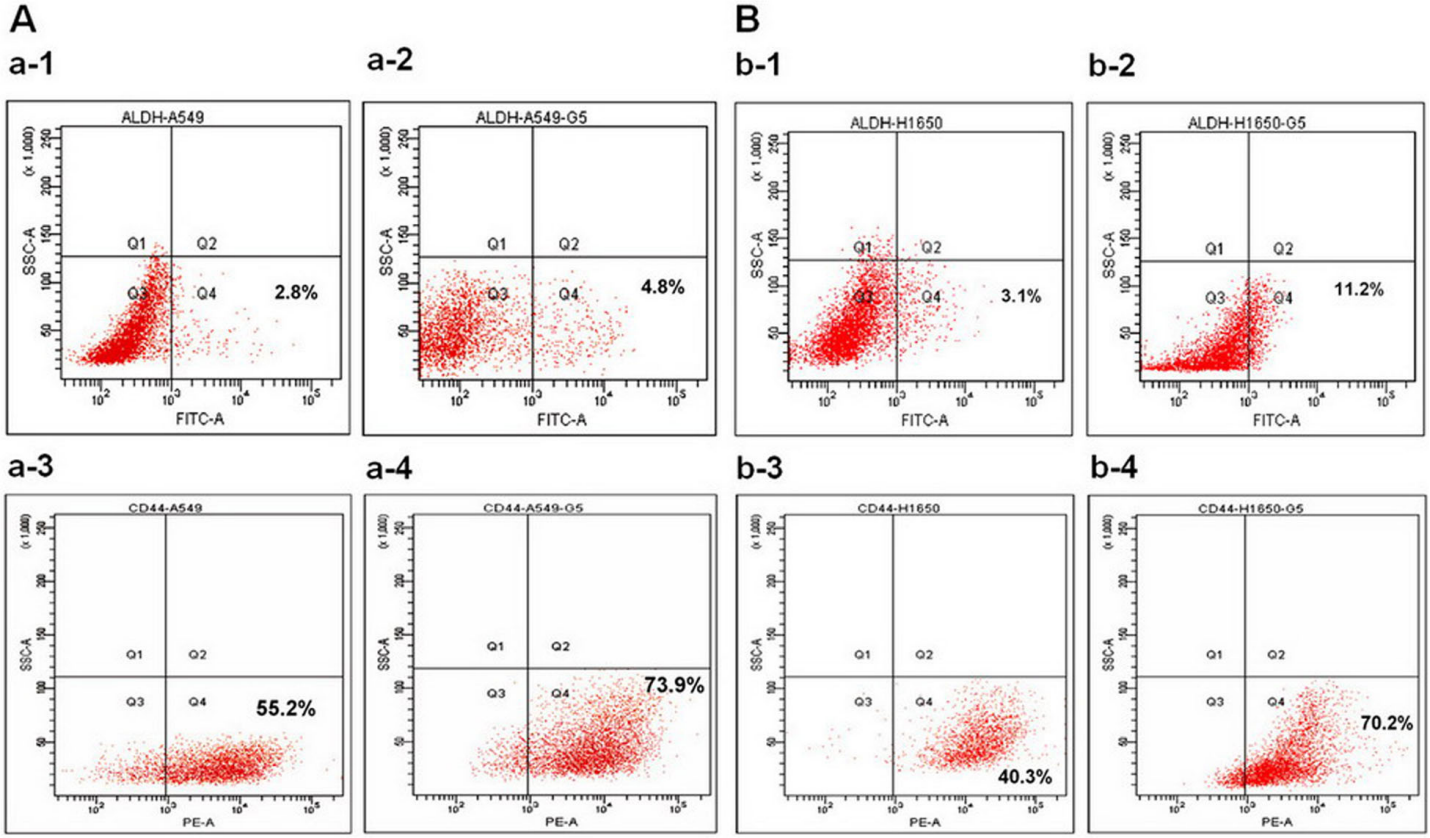
Detection of ALDH1A1 and CD44 expression of lung cancer cells treated with gefitinib by FCM as described in Methods. **a**. expression of ALDH1A1 (a-1 and a-2) and CD44 (a-3 and a-4) of A549 and A549GSC cells; **b**. expression of ALDH1A1 (b-1 and b-2) and CD44 (b-3 and b-4) of H1650 and H1650GSC cells. All experiments were performed in triplicate, and data are expressed as means±s.d. (*n* = 3). Error bars represent s.d. of replicate data points. *P < 0.05, compared to the control

As shown in Fig. 2, H1650GSC and A549GSC cells showed increased expression of ALDH1A1 and CD44, compared to the untreated cells (*P* < 0.05). The expression of ALDH1A1 and CD44 in A549GSC cells increased from 2.8 to 4.8% and from 55.2 to 73.9%, respectively (Fig. 2a); the expression of ALDH1A1 and CD44 in H1650GSC cells increased from 3.1 to 11.2% and from 40.3 to 70.2%, respectively (Fig. 2b). However, no EGFR T790M mutation in A549GSC and H1650GSC cells was detected using quantitative fluorescence PCR as described in methods (Supplementary Figure 1). These results indicate that resistance of A549GSC and H1650GSC cells to gefitinib could be associated with ALDH1A1^bright^/CD44^high^ but not associated with EGFR T790M mutation.

### ATRA reduces increased ALDH1A1 and CD44 expression of GSC cells

**Fig. 3:**
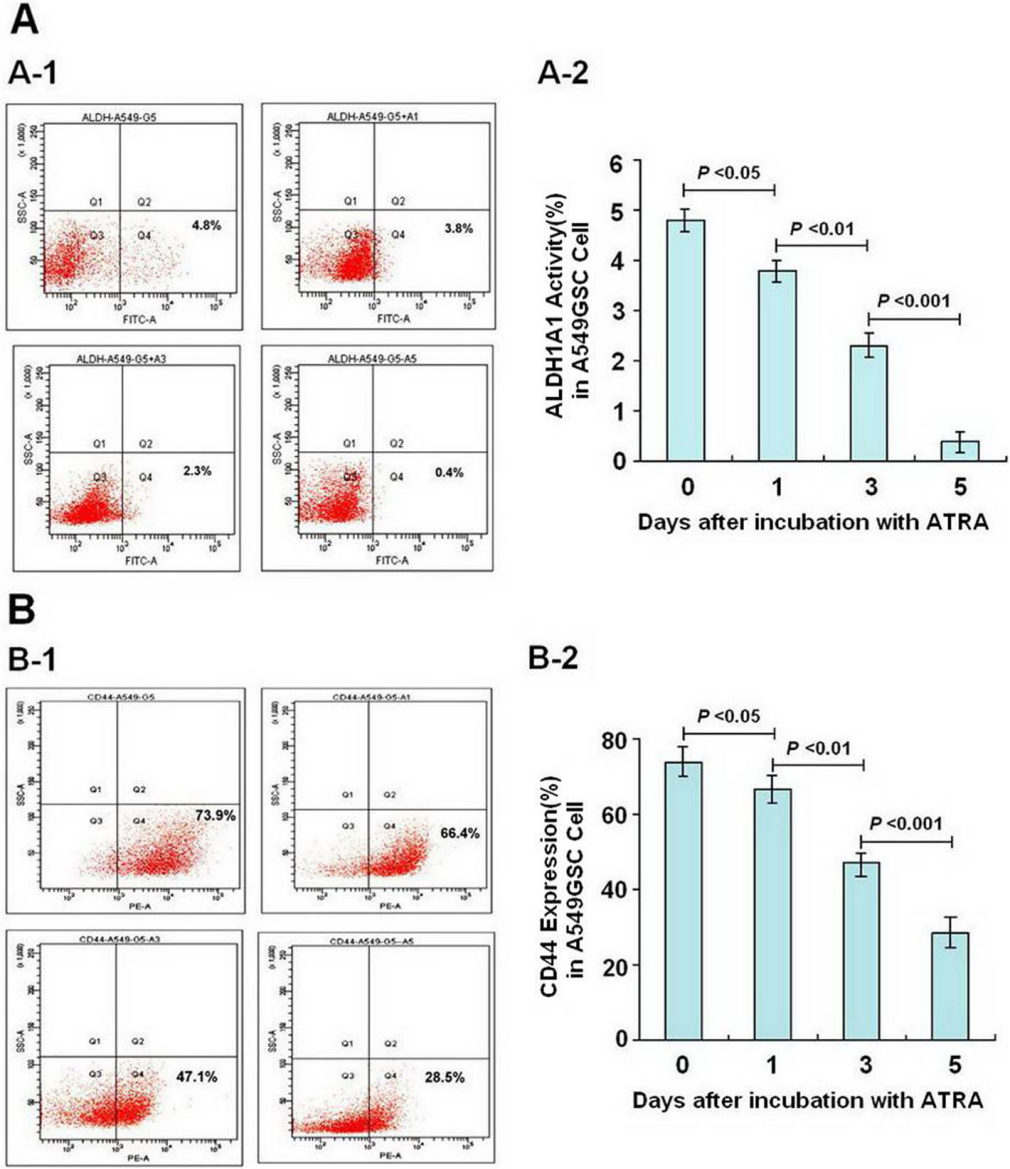
Detection of ALDH1A1 and CD44 expression of A549GSC cells incubation with ATRA for Day 1, 3, 5. **a** and **b**. Expression of ALDH1A1 (A-1 and A-2) and CD44 (B-1 and B-2) of A549GSC cells, respectively. All experiments were performed in triplicate, and data are expressed as means±s.d. (*n* = 3). Error bars represent s.d. of replicate data points. *P < 0.05, compared to the control

**Fig. 4:**
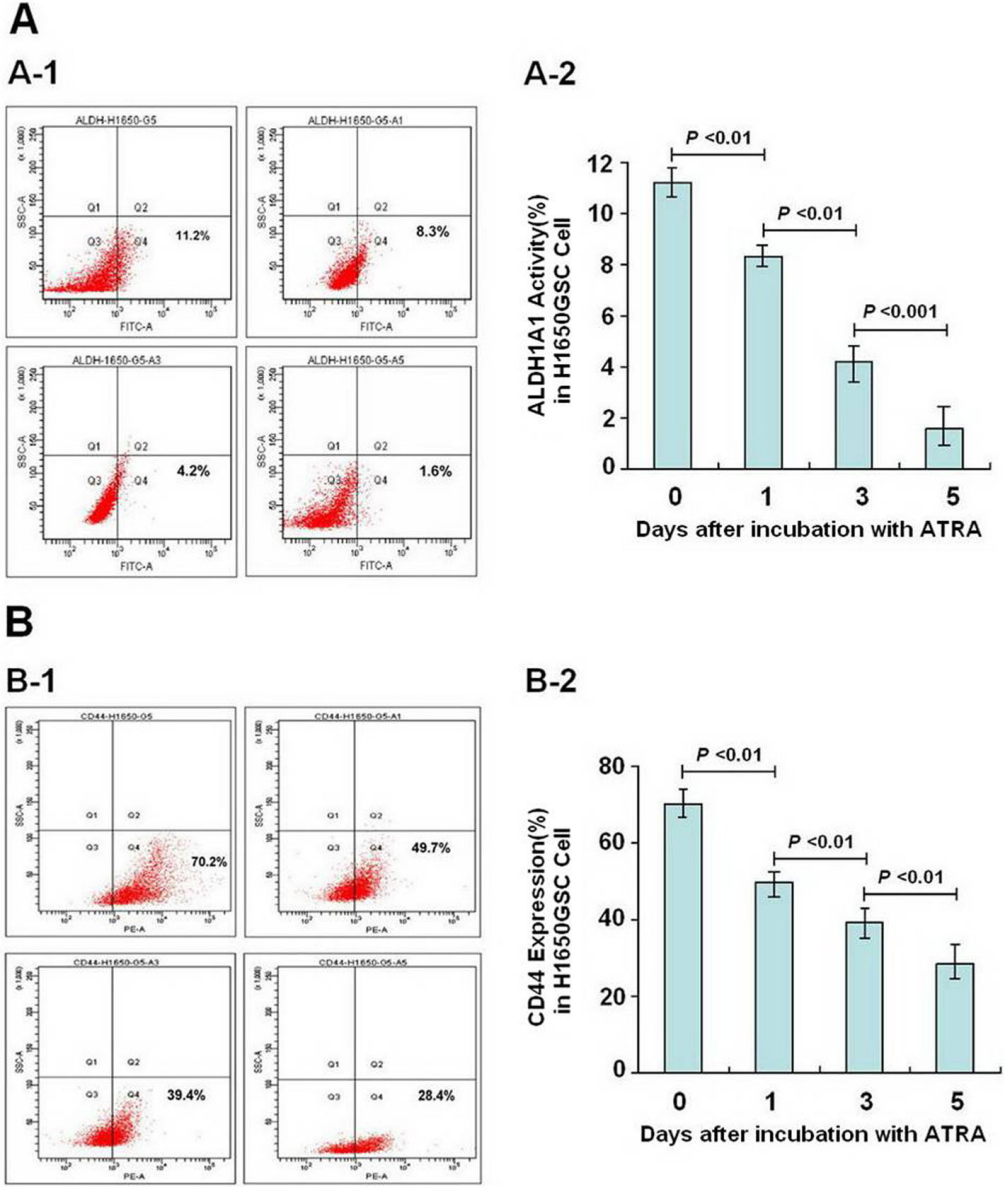
Detection of ALDH1A1 and CD44 expression of H1650GSC cells incubation with ATRA for day 1, 3, 5. **a** and **b**. Expression ALDH1A1 (A-1 and A-2) and CD44 (B-1 and B-2) of A549GSC cells, respectively. All experiments were performed in triplicate, and data are expressed as means±s.d. (*n* = 3). Error bars represent s.d. of replicate data points. *P < 0.05, compared to the control

As shown in Figs. 3 and 4, following incubation with ATRA for a different time, the expression of ALDH1A1 and CD44 of A549GSC and H1650GSC cells showed a significant decrease in a time-dependent manner. Approximate 12-fold and 2.6-fold decrease in expression of ALDH1A1 and CD44 (from 4.8 to 0.4% and from 73.9 to 28.5%) (Fig. 3a and b) and 7-fold and 2.5-fold (from 11.2 to 1.6% and 70.2 to 28.4%) (Fig. 4a and b) were observed in A549GSC and H1650GSC cells, respectively, compared to the control (both *P* < 0.01), and as shown in Supplementary Figure 2, ATRA could directly inhibit ALDH1A1 activity as compared to DEAB, suggesting that ATRA reduces propagation of A549GSC and H1650GSC cells showing ALDH1A1^bright^/CD44^high^.

## Discussion

CSC cell-mediated drug resistance of cancers is a major cause leading to the failure in cancer therapies. Resistance to molecular targeted drugs of NSCLC is a common characteristic of CSC cells. Among EGFR non-T790M mutation NSCLC cases, a small sub-population of cells within the tumor that possess stem-like properties come into being a more resistant phenotype during12 months after an initial response to the EGFR-TKIs in NSCLC [12]. Since potential relapse of NSCLC may occur due to the enrichment of CSCs following TKI initial therapy, the generation of more effective therapeutic interventions based on CSC cell-mediated resistance of NSCLC to EGFR-TKIs is an urgent requirement [17].

There is increasing evidence to show that ALDH1A1^bright^/CD44^high^ CSC-mediated EGFR-TKI resistance may be a major obstacle for EGFR-TKIs maintenance therapy of NSCLC. Loss of responsiveness to EGFR-TKIs in NSCLC with non-T790M EGFR mutation can be explained in terms of EGFR-TKI-resistant ALDH1A1^bright^/CD44^high^ CSC that evolutionally possesses drug resistance and is often referred to as a tumor-initiating cell and associated with EGFR-TKIs non-responder [12, 17, 28]. Recent studies showed that ALDH1A1^bright^ CSCs promote EGFR-TKI resistance in NSCLC [29, 30]. With respect to CD44, it has been reported that modulation of CD44 is detrimental to CSCs self-renewal and differentiation and NSCLC cells expressing CD44 are enriched for stem-like properties, suggesting that ALDH1A1^bright^/CD44^high^ CSCs linked to tumor progression and EGFR-TKI resistance is associated with a significantly poor prognosis factor in NSCLC [14, 31].

Differentiating CSCs may provide such an approach to modulating or converting the phenotypes of CSCs for sustained treatment response of NSCLC to EGFR-TKIs, although the mechanisms underlying CSCs contribution to resistance of NSCLC to TKIs remain unclear [32].

The ALDH-retinoic acid pathway plays an important role in differentiation of CSCs. It has been shown that treatment of lung adenocarcinoma A549 cell with ATRA led to the downregulation of ALDH1A1 [33].

Retinoic acid (RA) can reduce the ALDH activity and CD44 expression, thus affecting cell proliferation, cancer invasiveness and sensitivity to various chemotherapy drugs [24, 34, 35]. ALDH1A1 has been shown to convert/oxidize retinaldehyde into RA in several tissues, and to be one of the target proteins of ATRA [24]. Treatment with ATRA increased the C/EBP homologous protein (GADD153) and GADD153-CCAAT-enhancing binding protein-β (C/EBP-β) interaction resulting in a decreased cellular availability of C/EBP-β for binding to the Raldh1 CCAAT box and high ATRA levels inhibit Raldh1 gene expression by sequestering C/EBP-β through its interaction to GADD153 [36, 37]. CD44 expression was highly responsive to ATRA as it was down regulated following treatment, and ATRA treatment also resulted in decreased migration and invasion of cancer cells and promoted tumor regression by inducing differentiation [34].

Retinoids prevent the development of several tumors and enhance the efficacy of cytotoxic drugs such as cisplatin and docetaxel [38, 39]. Retinoids bind to specific nuclear receptors, which function as transcriptional regulators controlling the expression of numerous genes. The retinoid X receptors (RXRs) and retinoic acid receptors (RARs) are selectively expressed in ALDH^bright^ CSCs, indicating RA signaling mainly occurs via ALDH^bright^ CSCs of lung cancer, which provides a potential mechanism to selectively target CSCs [26, 40]. As shown in Figure S3, RA signaling is modulated by two classes of nuclear retinoid receptors, RARs and RXRs. Both RXRs and RARs interact with multiple co-activator and co-repressor proteins to promote increased cell stemness or cell differentiation. Retinoic acid showed feedback inhibition of the ALDH1 gene through RARα and C/EBP-β [41]. Specifically, RA signaling regulates ALDH via the binding of ATRA to RXR and RAR that transcriptionally control ALDH gene expression [38].

Loss of retinoid receptors expression happens frequently in the development of carcino- genesis and induction of resistance to apoptosis. The known effect of ATRA on differentiation of cells is mediated through RARβ. RARβ belongs to the nuclear receptor (NR) superfamily of transcription factors. Upregulation of RARβ within the drug-resistant cancer cells, which exhibits loss of RARβ expression, has been shown to increase the susceptibility of cells to apoptosis induced by chemotherapeutic agents. Activation of RARs or RXRs contributes to induction of RARβ, growth inhibition and apoptosis by retinoids. It evidenced that the therapeutic anti-CSC and proapoptotic effects of ATRA are dependent on receptor class-selective retinoids and the expression of RARβ plays a role in mediating retinoid response in NSCLC cells [38, 41]. The loss of RARβ might contribute to enhanced cancer stemness and the apoptosis resistance of CSCs to gefitinib in NSCLC cells. ATRA can induce the apoptosis of NSCLC CSCs through activation of RARβ and its ability to down-regulate the CSCs markers in lung cancer cells [42, 43]. The expression of RARβ as well as RXRβ was reported to be downregulated in NSCLC, which enabled the cancer cell to evade apoptosis [44]. The RARβ is also known as tumor suppressor and the major target gene of retinoid action, and an enhanced level of RARβ protein exhibited its growth inhibitory action of lung cancer cells [44, 45]. RARβ can mediate retinoid action in lung cancer cells by promoting apoptosis. However, a fundamental question that remains unanswered is how ATRA and RARβ trigger apoptosis in lung cancer cells. Studies showed the overexpression of RARβ was accompanied by an increase in c-Myc and Bax but not TP53 protein expression and associated with an increase in the Bax/Bcl-2 ratio, and that ATRA enhanced G1 growth arrest, up-regulated p21and p27 and downregulated cyclin D1. These data suggest that the expression of RARβ is positively associated with ATRA-induced apoptosis and growth inhibition in lung cancer cells. It has been shown that RA inhibits EGFR expression at the transcriptional level by targeting the EGFR promoter leading to inhibition of lung cancer cell growth and arrests EGFR-TKI resistant NSCLC cells in the G0/G1 phase of the cell cycle by altering the expression of GATA-binding factor 6 (GATA6) and inhibits the activation of two important pathways involved in lung cancer progression namely EGFR and Wnt signaling to overcome TKI resistance [46]. Combinatorial treatment of retinoids with EGFR-TKIs drugs in EGFR-TKIs resistance lung cancer cells promotes the activation of GATA6 and then inhibits the activation of EGFR/Wnt signaling pathways and favors the association of RXR, RARβ, and cellular retinoic acid binding protein-2 (CRABP2). This complex inhibits the proliferation and promotes the differentiation of lung tumor cells via inhibiting activating protein-2 (AP-2), which result in re-sensitization of EGFR-TKIs resistant lung cancer cells [47].

In this study, a short-term gefitinib treatment was used to enrich A549GSCs and H1650GSCs. FCM assay showed that A549GSCs and H1650GSCs have a significant increase in proportions of ALDH1A1bright/CD44high cells (Fig. 2), and we further confirmed that these ALDH1A1bright/CD44high GSCs exhibit increased IC50 values for gefitinib compared to that of their respective parental cells (Table 1 and Fig. 1a and b), and are involved in CSCs but not in EGFR T790M-mediated gefitinib resistance (Supplementary Figure 1), suggesting that ALDH1A1bright/CD44high CSCs in NSCLC/ADC contribute to resistance to gefitinib. Interestingly, treatment with ATRA significantly reduced ALDH1A1 and CD44 expression of A549GSCs and H1650GSCs, and their IC50 values for gefitinib, thus returning to sensitization to gefitinib (Table 1 and Fig. 1a and b). ALDEFLUOR assay also showed that ATRA can directly inhibit ALDH1A1 activity in a concentration dependent manner (Supplementary Figure 2). These results showed that in contrast to the known tendency of EGFR-TKIs, such as gefitinib, to target the non-stem-like ALDH1A1-negative cell population, ATRA can modulate the ALDH1A1bright/CD44high cell population in NSCLC/ADC. Therefore, the synergistic antitumor effect of ATRA in combination with gefitinib might be a promising therapeutic strategy to circumvent CSC-mediated resistant NSCLC/ADC.

## Conclusions

Our findings indicate that combinatorial treatment of ATRA with gefitinib could reduce CSC-mediated resistance by down-regulating expression of ALDH1A1 and CD44 and potentiate the anti-tumor effect of gefitinib in NSCLC/ADC.

## Supplementary information

**Additional file 1 **: **Figure S1.** Detection of EGFR mutation of A549GSC and H1650GSC cells by ARMS-qPCR as described in Methods**.*A.*** EGFR mutation of A549 cells before (A-1) and after (A-2) treatment with gefitinib; ***B.*** EGFR mutation of H1650 cells before(B-1) and after(B-2) treatment with gefitinib.

**Additional file 2 **: **Figure S2.** Direct inhibitory effect of ATRA on ALDH1A1 activity in GSC cells (FITC AUC) by ALDEFLUOR assay as described in Methods**.*A*.** ALDH1A1 Activity of A549GSC (A-1, A-2 and A3); ***B*.** ALDH1A1 Activity of H1650GSC (BA-1, B-2 and B3).

**Additional file 3 **: **Figure S3.** A potential mechanism by which ATRA regulates the response of lung cancer stem cells to gefitinib. ATRA binds and activates RARβ complex and related signaling molecules. The interaction of C/EBP homologous protein (GADD153) with GADD153-CCAAT-enhancing binding protein-β (C/EBP-β) results in a decreased cellular availability of C/EBP-β for binding to the Raldh1 CCAAT box, and high ATRA levels can sequester interaction of C/EBP-β with GADD153 to suppress expression of Raldh1 gene.

## Data Availability

Please contact author for data requests.

## Abbreviations

EGFR-TKD: Epidermal growth factor receptor- tyrosine kinase domain
TKIs: Tyrosine kinase inhibitors
ATRA: All-trans retinoic acid
NSCLC/ADC: Non-small cell lung adenocarcinoma
ALDH1A1: Aldehyde dehydrogenase 1 family member A1
CD44: Cluster of differentiation 44
FCM: Flow cytometry
IC50: Half maximal inhibitory concentration
NSCLC: Non-small cell lung cancer
EGFRm: Mutant epidermal growth factor receptor
ATP: Adenosine triphosphate
EGFR-TKIs: EGFR tyrosine kinase inhibitors
ADC: Adenocarcinoma
EGFRWT: Wild-type EGFR
SCLC: Small cell lung cancer
MET: Cellular-mesenchymal to epithelial transition factor
ERBB2: Erythroblastic leukemia viral oncogene homolog 2
PIK3CA: Phosphatidylino-sitol 3-kinases, catalytic, alpha polypeptide
EMT: Epithelial to mesenchymal transition
CSC: Cancer stem cell-like cell
RAs: Retinoic acids
RR: Response rate
PFS: Progression-free survival
APL: Acute promyelocytic leukemia
CTCCCAS: The committee on type culture collection of Chinese academy of sciences
FBS: Fetal bovine serum
EDTA: Ethylenediaminetetraacetic acid
TAE: Tris/Acetate/EDTA
PCR: Polymerase chain reaction
GSC: Gefitinib surviving cell
TME: Tumor microenvironment
GADD153: C/EBP homologous protein, CHOP
EBPb: CCAAT-enhancing binding protein-B
RARs: Retinoic acid receptors
GATA6: GATA-binding factor 6
CRABP2: Cellular retinoic acidbinding protein-2
DEAB: Diethylaminobenzaldehyde
AP-2: Activating protein-2
