# Supplementary figures and images for: All-trans retinoic acid reduces cancer stem cell-like cell-mediated resistance to gefitinib in NSCLC adenocarcinoma cells
Source: BMC Cancer. 2020 Apr 15;20:315. doi: 10.1186/s12885-020-06818-0 (PMC7161137; doi:10.1186/s12885-020-06818-0)

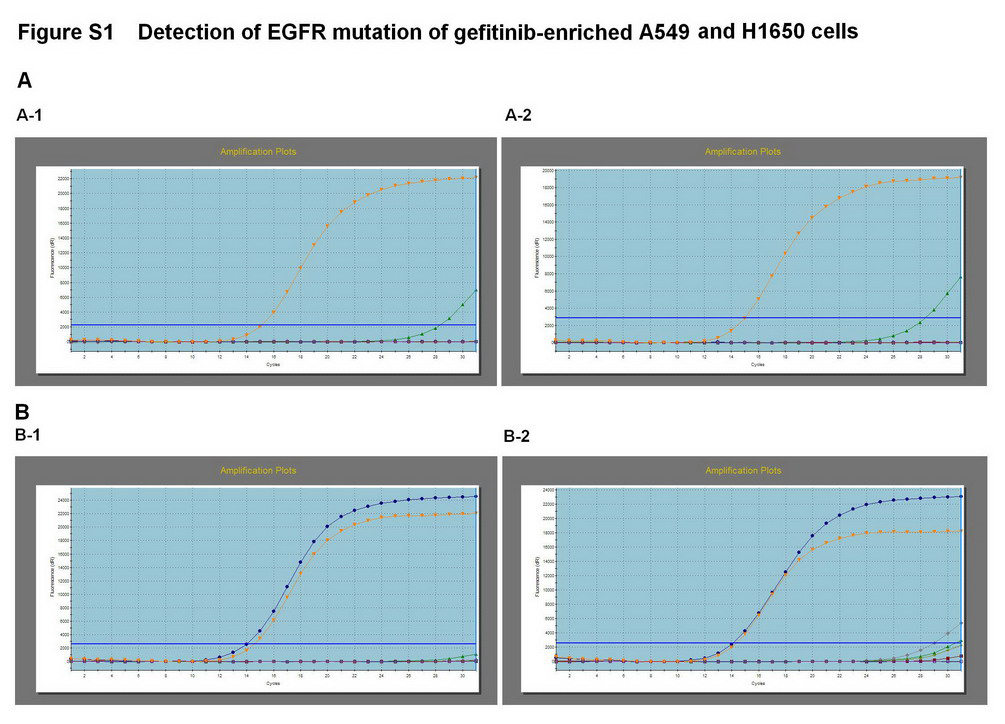

Supplement: Supplementary file 1 — Additional file 1 : Figure S1. Detection of EGFR mutation of A549GSC and H1650GSC cells by ARMS-qPCR as described in Methods.A. EGFR mutation of A549 cells before (A-1) and after (A-2) treatment with gefitinib; B. EGFR mutation of H1650 cells before(B-1) and after(B-2) treatment with gefitinib. [file 12885_2020_6818_MOESM1_ESM.jpg]

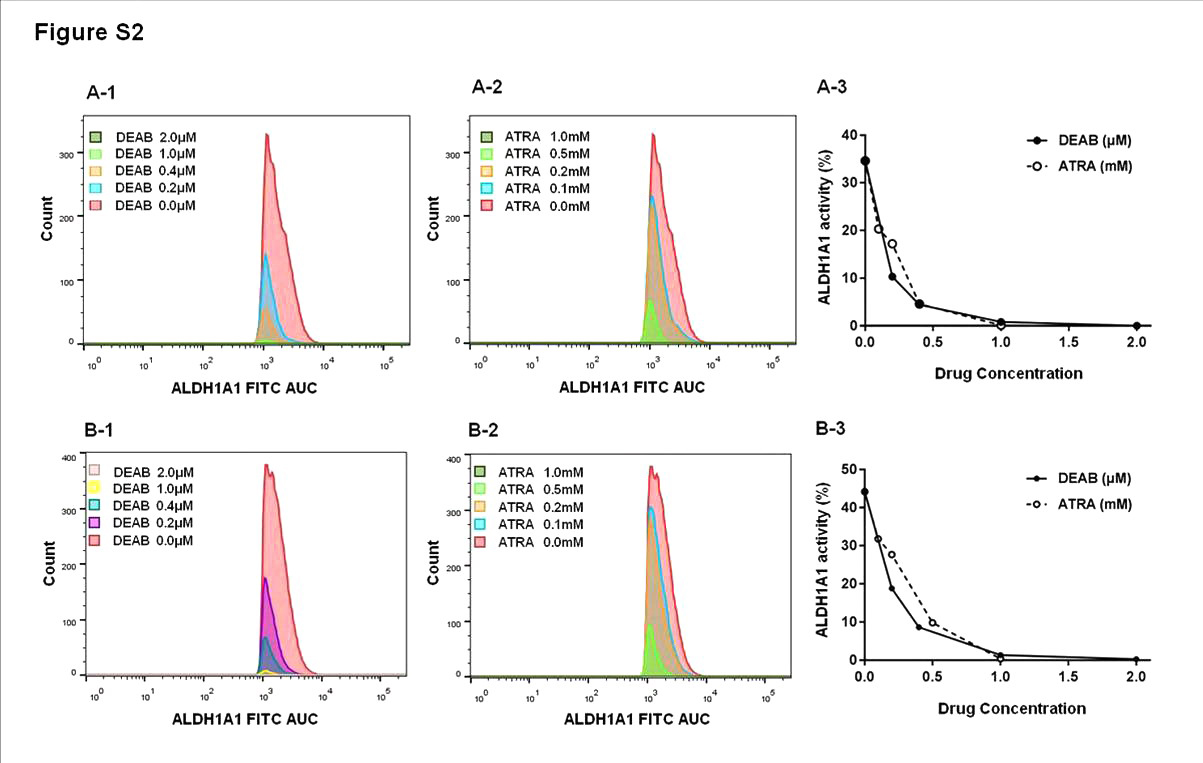

Supplement: Supplementary file 2 — Additional file 2 : Figure S2. Direct inhibitory effect of ATRA on ALDH1A1 activity in GSC cells (FITC AUC) by ALDEFLUOR assay as described in Methods.A. ALDH1A1 Activity of A549GSC (A-1, A-2 and A3); B. ALDH1A1 Activity of H1650GSC (BA-1, B-2 and B3). [file 12885_2020_6818_MOESM2_ESM.jpg]

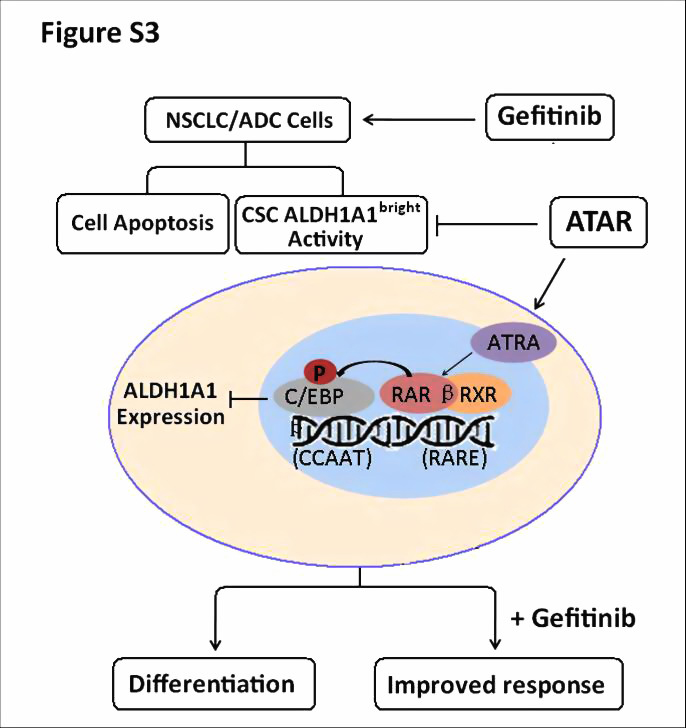

Supplement: Supplementary file 3 — Additional file 3 : Figure S3. A potential mechanism by which ATRA regulates the response of lung cancer stem cells to gefitinib. ATRA binds and activates RARβ complex and related signaling molecules. The interaction of C/EBP homologous protein (GADD153) with GADD153-CCAAT-enhancing binding protein-β (C/EBP-β) results in a decreased cellular availability of C/EBP-β for binding to the Raldh1 CCAAT box, and high ATRA levels can sequester interaction of C/EBP-β with GADD153 to suppress expression of Raldh1 gene. [file 12885_2020_6818_MOESM3_ESM.jpg]
